# Lung Ultrasound Is Accurate for the Diagnosis of High-Altitude Pulmonary Edema: A Prospective Study

**DOI:** 10.1155/2018/5804942

**Published:** 2018-10-01

**Authors:** Weibo Yang, Yuliang Wang, Zewu Qiu, Xuewen Huang, Maoxia Lv, Bin Liu, Dingzhou Yang, Zhenhan Yang, Tingshan Xie

**Affiliations:** ^1^Department of High Altitude Disease, Xizang Military General Hospital, Lhasa, China; ^2^Digestive System Department of Affiliated 307 Hospital, Academy of Military Science of the People's Liberation Army, Beijing, China; ^3^Department of Ultrasound, Xizang Military General Hospital, Lhasa, China; ^4^Department of Radiology, Xizang Military General Hospital, Lhasa, China

## Abstract

**Objective:**

The aim of this study was to assess the diagnostic accuracy of lung ultrasonography (LUS) for high-altitude pulmonary edema (HAPE).

**Background:**

LUS has proven to be a reliable tool for the diagnosis of pulmonary diseases, including pneumonia, acute respiratory distress syndrome (ARDS), and pneumothorax. LUS also has potential for the diagnosis of HAPE. However, the actual diagnostic value of LUS for HAPE is still unknown. Our objective was to determine the feasibility of using LUS for the diagnosis of HAPE.

**Materials and Methods:**

A prospective clinical research study of adult HAPE patients was conducted. LUS and chest X-ray (CXR) were performed in patients with suspected HAPE before and after treatment, and pulmonary moist rales were recorded concurrently. The diagnostic value of LUS, CXR, and moist rales for HAPE (i.e., their sensitivity, specificity, and positive and negative predictive values) were assessed, and the results were compared. The gold standard was the final diagnosis.

**Results:**

In total, 148 patients were enrolled in the study, 126 of which were diagnosed with HAPE (85.14%). Before treatment, the diagnostic accuracy of LUS for HAPE was as follows: sensitivity, 98.41% (95% confidence interval (CI) 100.60–96.23%); specificity, 90.91% (95% CI 102.92–78.90%). LUS had higher sensitivity (0.98 vs. 0.81, *P* < 0.01 using the McNemar test) than moist rales for the diagnosis of HAPE. LUS also had higher sensitivity than CXR (0.98 vs. 0.93, *P* < 0.05 using the McNemar test). After treatment, LUS was consistent with CXR in 96.55% of HAPE patients, and the concordance between LUS and CXR was high (*k* statistic = 0.483 *P* ≤ 0.001; 95% CI −0.021 to −0.853).

**Conclusion:**

The results indicate that LUS is a reliable method for the diagnosis and surveillance of HAPE. This trial is registered with Chinese Clinical Trial Registry (No. ChiCTR-DDD-16009841).

## 1. Introduction

High-altitude pulmonary edema (HAPE) is an acute and severe altitude disease, and its primary characteristic is pulmonary edema induced by hypoxic environment [1, 2].

Until recently, the most valuable ancillary diagnostic tool for HAPE was chest radiography. However, X-ray examination may be unavailable or inconvenient in remote locations or under emergency conditions. Frequent exposure to radiation is also problematic, especially for pregnant women or children. Thus, a more convenient and reliable tool is needed for the diagnosis of HAPE.

Lung ultrasound (LUS) is a reliable diagnostic tool for pulmonary diseases [3]. If the fluid in the pulmonary parenchyma or alveoli increases, vertical echoic shadowing from the pleural line to the bottom of the screen (B-line) should appear in LUS [4]. The number and density of the B-lines can be used for the surveillance of pulmonary edema [5]. Previous studies have indicated that ultrasonography might be useful in HAPE patients [6–8]. However, no studies have investigated whether ultrasound is superior to routine physical examinations or chest X-ray (CXR) for the diagnosis of HAPE. Therefore, the actual diagnostic value of LUS for HAPE is unknown. Our study attempts to answer this question.

**Figure 1:**
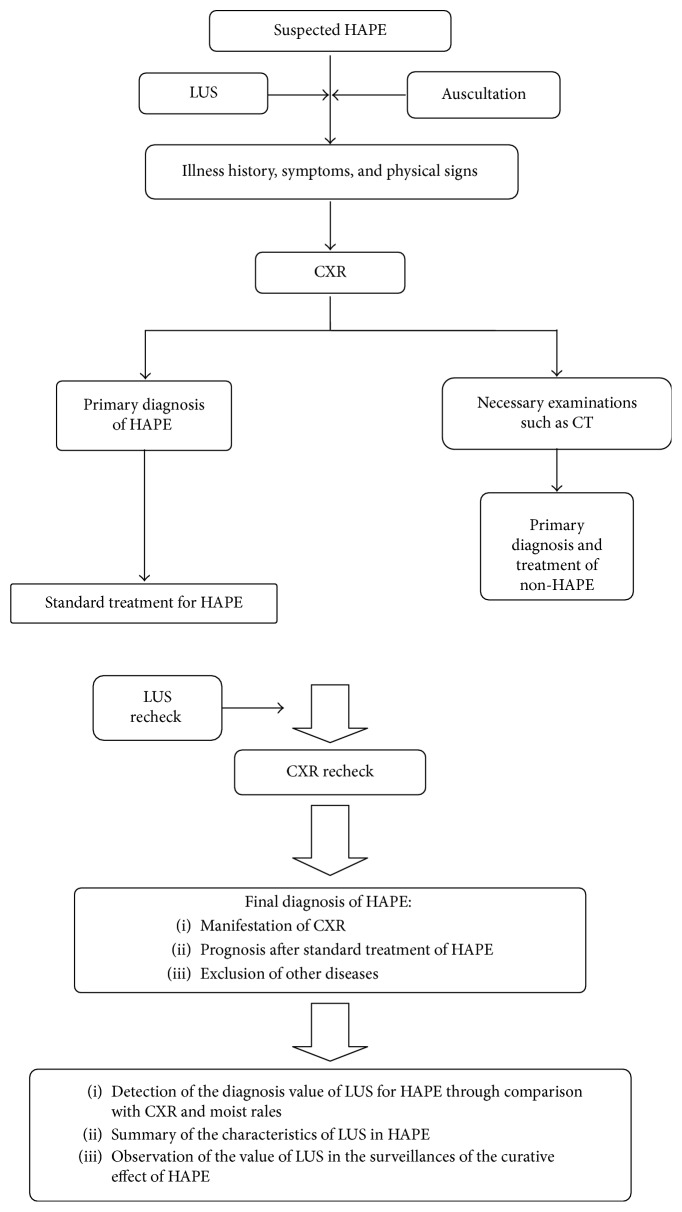
The study design.

The primary aim of our study was to determine the sensitivity and specificity of prehospital lung ultrasound for HAPE. The diagnosis at hospital discharge was used as the gold standard. The secondary aim was to assess the surveillance value of LUS for assessing the effect of treatment in HAPE patients. The design is showed in Figure 1.

### 1.1. Setting

A prospective study was conducted in the Department of High Altitude Diseases of Tibet Military General Hospital, China. This research was approved by the Ethics Committee of Tibet Military General Hospital (KY20160501).

### 1.2. Participants

We studied a consecutive sample of adult patients admitted to the Department of High Altitude Diseases for suspected HAPE. All patients were older than 10 years of age and were not pregnant.

Patients who met all of the following criteria were suspected of having HAPE: in a plateau location or higher altitude district within one week prior, cough, chest tightness, dyspnea, and obvious cyanosis. The exclusion criteria were the following: consciousness disorders; patients who were unable to follow the research plan and sign the consent form; a history of chronic pulmonary diseases, including interstitial lung disease, COPD, or other parenchymal lung disease; and a history of cardiac disease (ischemia, arrhythmia, valve dysfunction, or cardiomyopathy).

After enrollment, all patients were admitted to the high-altitude disease ward and underwent LUS, CXR, and a physical examination. Patients diagnosed with HAPE initially accepted the standard treatment for HAPE, and the non-HAPE patients accepted the corresponding treatment. If the diagnosis was changed during treatment, the program of treatment was changed according to the clinical needs.

## 2. Materials and Methods

**Figure 2:**
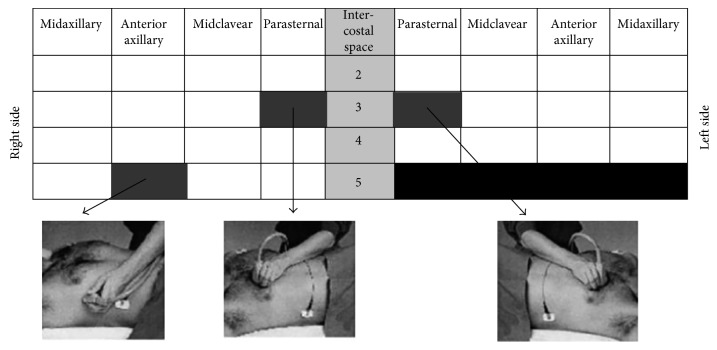
Lung ultrasound scanning for B-lines (cited from L Gargani et al., “Ultrasound lung comets for the differential diagnosis of acute cardiogenic dyspnea: a comparison with natriuretic peptides,” *European Journal of Heart Failure*, 10 (2008) 70–77).

LUS was performed using the LOGIQ 7 Pro Digital Premium Ultrasound System (GE Healthcare, USA) with a convex 3.5C broadband 2.0–5.0 MHz probe. Scanning was performed with the patient in the supine position, and the probe was placed perpendicular to the chest wall over the intercostal spaces. Both lungs were scanned along the parasternal, midclavicular, anterior axillary, and midaxillary lines. For the left lung, the scanning range was from the second to the fourth intercostal spaces, and for the right lung, the scanning range was from the second to the fifth intercostal spaces (Figure 2); in total, 28 scanning points served as the reference [9]. The number of B-lines was evaluated at each point. At each scanning site, the B-lines were counted from zero to ten. Zero was defined as a complete absence of B-lines in the investigated area, whereas a full white screen in a single scanning site was considered to represent 10 B-lines. At times, the B-lines tended to be confluent and difficult to count. In these situations, we determined the percentage of the screen occupied by the B-lines and divided this percentage by ten. The B-line score was the total number of B-lines at all 28 scanning points, as previously described [10]. A B-line score ≤5 was considered negative for HAPE [4, 9]. In contrast, a B-line score >5 was considered positive for HAPE.

After the participants were admitted to the Department of High Altitude Diseases, a lung ultrasound was immediately performed prior to treatment. An experienced physician performed the LUS and determined the imaging diagnosis together with the B-line score. The LUS images were stored in DVD format.

In order to assess the reliability of LUS, intraobserver variability and interobserver variability were detected later. After the patients were discharged from the hospital, all identifying information was removed from the LUS images; then, the previous physician and another experienced physician read the handled images in a blinded manner to assess the intra and interobserver variability. Throughout the process, the two physicians were both blinded to any X-ray findings and clinical information.

CXR in the posteroanterior view was performed within 1 hour following ultrasound examination. Similar to LUS, CXR was performed before treatment and stored in DVD format. After the patients were discharged from the hospital, all identifying information was removed from the CXR images; then, two experienced radiologists read the images in a blinded manner. The first radiologist determined a diagnosis based on the X-ray images, and the second radiologist determined a radiologic score related to the extravascular lung fluid according to the reference [11]. The radiologists were blinded to any clinical information and the LUS results.

The positive CXR results indicating HAPE were defined according to “Diagnostic Criteria of High Altitude Disease in China”: a patchy or cloudy infiltrate shadow centered on the hila and radiated to one or two sides of the lung fields [12].

For all patients, LUS and CXR were performed again after treatment. Reexaminations were performed on the day of hospital discharge. The CXR and LUS images obtained after treatment were read by the same physicians who read the images before treatment.

An independent experienced doctor who was blinded to any clinical information (including the LUS and CXR) recorded moist rales before treatment and on the day of discharge. The moist rales were auscultated in the same position that was scanned in the LUS and were recorded as positive or negative for each person.

Oxygen saturation was measured using a Fingertip pulse oximeter (ver1.0, the EMC of this product complies with the IEC60601-1-2 standard, Academy of Military Medical Sciences) before treatment and on the day of discharge.

All results of clinical research mentioned above, including LUS CXR and moist rales, were only used for clinical research and blinded to clinical doctors.

### 2.1. Standard Treatment Protocol for HAPE

(1) Oxygen was delivered. (2). Aminophylline and/or dexamethasone were injected intravenously. The altitude where the remedy was performed for all patient s was 3700 m.

### 2.2. Final Diagnostic Criteria for HAPE

Participants who met all of the following criteria were diagnosed with HAPE.

(1) The CXR showed typical signs of pulmonary edema before treatment; (2) noted improvements in the pathogenic conditions and CXR images occurred rapidly after standard treatment for HAPE; and (3) other diseases were excluded.

The diagnosis and treatment were performed by experienced doctors who were blinded to the clinical research results.

### 2.3. Discharge Criteria for HAPE

Patients who met all of the following criteria were discharged from the hospital.

(1) Typical symptoms (including cough, sputum, and chest tightness) were completely relieved; (2) abnormal physical signs, including cyanosis and moist rales, vanished completely; and (3) the patients received the standard treatment for HAPE for at least 48 hours.

### 2.4. Statistical Analysis

The diagnostic accuracy of the LUS, CXR, and moist rales before treatment for the diagnosis of HAPE was calculated. The sensitivity, specificity, positive predictive value (PPV), negative predictive value (NPV), positive likelihood ratio (PLR), negative likelihood ratio (NLR), and 95% confidence intervals (CIs) were calculated using standard formulas. The discrepancies in sensitivity and specificity between LUS and CXR and between LUS and moist rales were calculated using McNemar's Chi-square test. The final diagnosis was the reference test for all calculations.

The intra- and interobserver variability of the LUS was measured by calculating intraclass correlation coefficient (ICC).

The concordance between the LUS and CXR of HAPE patients after treatment was also calculated. The result was reported using the statistic.

ROC curves with LUS score of all participants before treatment was calculated either.

Linear regression analysis was used to assess whether the independent variable B-line score had significant impact on the dependent variable oxygen saturation before treatment.

The McNemar, ICC, ROC curves, Linear regression analysis, and *k* statistical analyses were performed using SPSS 22.0 software. A *P* value of 0.05 was considered significant.

## 3. Results

From November 1, 2016, to March 31, 2017, 148 suspected HAPE patients, including 9 females and 139 males, were enrolled in the study. Overall, 126 cases were diagnosed with HAPE, and 22 patients received a different diagnosis. Of the patients who were not diagnosed with HAPE, 1 case had bronchitis, 4 cases had acute upper respiratory infections, and 17 cases were diagnosed with acute mild high-altitude disease (Tables 1 and 2).

**Figure 3:**
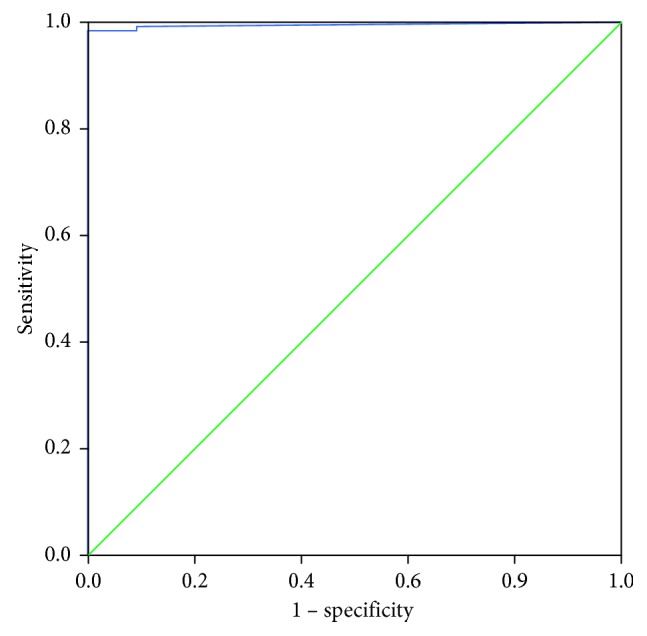
ROC curve with LUS score of all participants before treatment. The best critical value of B-line score for the diagnosis of HAPE was 6.5 and the ROC curve's area was 0.995.

Of the 126 HAPE patients, 124 cases showed positive LUS results (B-line score >5). In the non-HAPE patients, only 2 cases diagnosed with acute mild high-altitude disease showed positive LUS results (B-line score >5). In all participants, no other abnormal signs except B-lines were detected on the LUS. ROC curve with LUS score was also established. The best critical value of B-line score for the diagnosis of HAPE was 6.5 and the ROC curve's area was 0.995 (Figure 3). The result was close to the previous references [4, 9].

The intra- and interobserver variability in the B-line scores from the ultrasound images before treatment was assessed by 2 independent observers. The assessments had high ICC indicating excellent repeatability. The within and between observers ICC were 0.995 (95% CI 0.993–0.996) and 0.981 (95% CI 0.974–0.986) separately.

Moist rales were recorded in only 102 HAPE patients before treatment. Additionally, moist rales were recorded for 3 non-HAPE patients before treatment (Table 3).

The sensitivity, specificity, PPV and NPV of LUS, CXR, and moist rales before treatment were calculated and compared with the final diagnosis (Table 4). The differences in the sensitivity and specificity were estimated and tested considering the matched-pairs design (Table 3). LUS had a higher sensitivity (0.98 vs. 0.81, *P* < 0.01 using McNemar's test) than moist rales for the diagnosis of HAPE. LUS also had a higher sensitivity than CXR (0.98 vs. 0.93, *P* < 0.05 with McNemar's test). The results showed that LUS was superior to CXR and moist rales for the diagnosis of HAPE. The comparison of clinical and LUS characteristics between HAPE+ and HAPE− patients before treatment was also shown in results (Table 1).

Of the 126 cases diagnosed with HAPE, 10 patients were not reassessed by CXR at discharge. We compared the LUS and CXR results at discharge: of the 116 HAPE patients who finished the clinical study, 110 cases had negative results on LUS and CXR; 2 cases had positive results on both LUS and CXR; 1 case had negative results on CXR but positive results on LUS; and 3 patients had negative findings on LUS and positive CXR results (Table 5). The concordance between LUS and CXR was high (*k* statistic = 0.483 *P* ≤ 0.001; 95% confidence interval −0.021 to −0.853).

**Figure 4:**
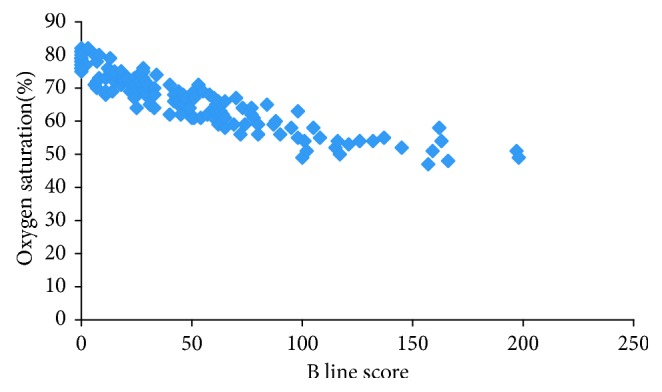
Distribution of oxygen saturation and B-line score in all participants (adjusted R2 for B-line score vs. oxygen saturation = 0.819; *P*=0.000). A scatterplot graph generated by Excel 2016 was provided to describe the relationship between B-line score and oxygen saturation.

Before treatment, B-line score was correlated with oxygen saturation closely (adjusted *R*2=0.819; *P*=0.000) (Figure 4).

We did not observe any adverse events related to LUS examination in this study.

## 4. Discussion

HAPE is mainly characterized by a large amount of exudation in the pulmonary alveoli [13]. Thus, HAPE may have ultrasound manifestations similar to those of other types of pulmonary edema. Previous related studies support this hypothesis.

Fagenholz et al. observed that HAPE patients had higher B-line scores than healthy subjects [6]. B-line score could also be used to monitor the therapeutic effects in HAPE patients [7]. Lorenza Pratali observed an increased B-line score even in patients with subclinical HAPE [8]. These studies indicated that ultrasonography might be valuable for the diagnosis and treatment of HAPE. However, these studies only enrolled a small number of participants (11 subjects in Fagenholz, 18 subjects in Lorenza and 10 subjects in Peter), and CXR and auscultation were not adopted as references for the diagnosis; thus, none of the studies could demonstrate the actual diagnostic value of LUS for HAPE. Through comparisons between LUS and routine examinations, including auscultation or CXR, our study evaluated the diagnostic value of LUS for HAPE for the first time.

**Figure 5:**
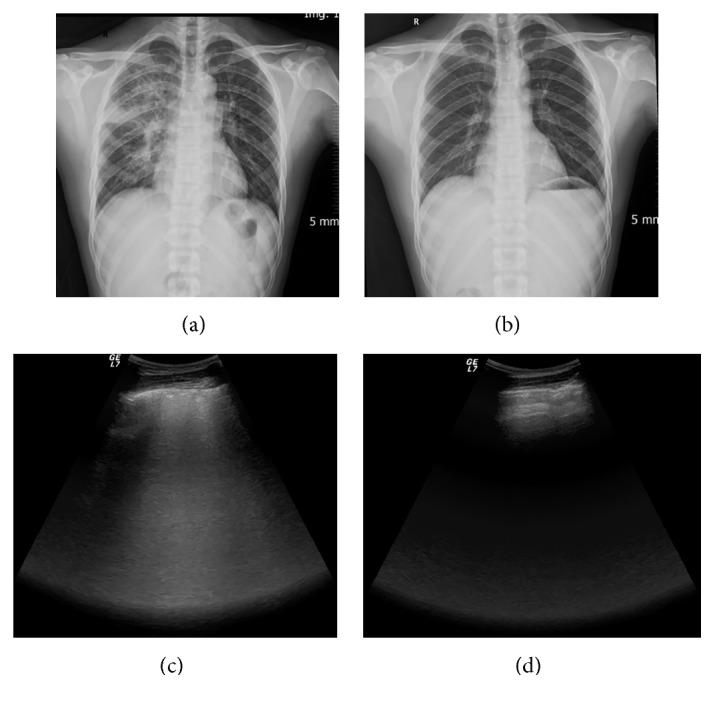
Characteristic manifestations of HAPE on LUS. Patients showed intensive B-lines. The B-lines vanished along with absorption of the pulmonary edema. Typical images from one HAPE patient: (a) CXR before treatment: large flocculent infiltrates in the right lung; (b) CXR after treatment: pulmonary edema was entirely absorbed; (c) LUS before treatment: multiple B-lines; (d) LUS after treatment: normal image.

**Figure 6:**
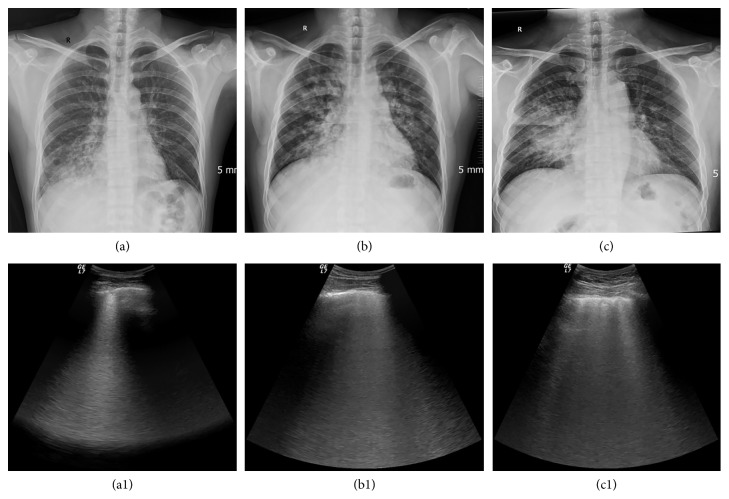
HAPE had multiple manifestations on CXR, whereas the LUS manifestations were only intensive B-lines. (a) Flake and trabs like shadow in inferior lobe of right lung; (a1), corresponding LUS image; (b) butterfly-shaped shadow; (b1), corresponding LUS image; (c) large infiltrates involving the entire lobar; (c1), corresponding LUS image.

Our experimental results were in accordance with previous studies. The main HAPE findings on LUS were multiple B-lines (Figure 5). Severe HAPE appears as a “white lung” phenomenon (Figures 5 and 6). In HAPE patients, the findings on LUS were unique and stable; in other words, apart from the intensive B-lines, no other findings were noted, including lung consolidation, air bronchogram, abnormal pleural line sliding, pleural effusion, or pleural thickening. Compared to the unique and simple signs on LUS, the X-ray findings in HAPE patients included multiple signs, such as flocculent, slabby, trabs, butterfly aliform, or ground glass shadows (Figure 6). Additionally, our results showed that LUS had a higher sensitivity than CXR for the diagnosis of HAPE. For doctors who lack experience diagnosing HAPE, LUS is simple and easier to grasp than X-ray examination.

**Figure 7:**
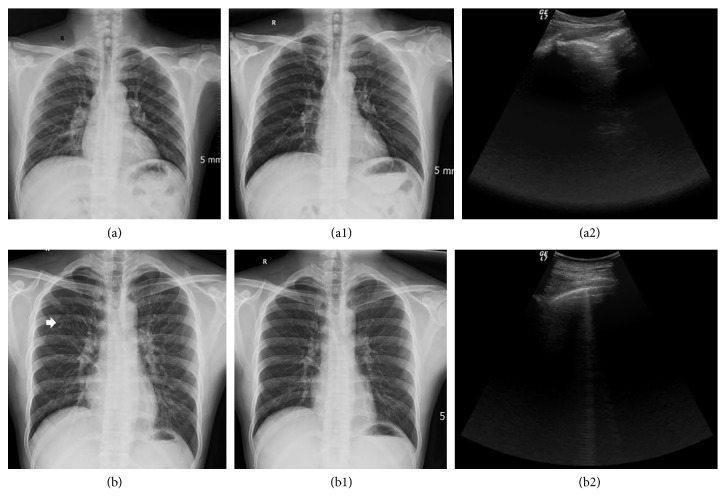
Examples of HAPE patients with negative manifestation of LUS. (a) CXR before treatment: enlarged hilar shadows can be observed; (a1) corresponding CXR after treatment: the image returned to normal; (a2) corresponding LUS before treatment: the images were normal, and no B-lines were detected; (b) CXR before treatment: densified hilar shadows and small patch shadows can be observed in the upper left lung (white arrow); (b1) corresponding CXR after treatment: the image returned to normal; (b2) corresponding LUS before treatment: only a few B-lines (B-line score = 2) were detected.

Only 2 diagnosed cases of HAPE were negative on LUS. These cases all showed a small amount of pulmonary edema on the CXR, as evidenced by an enlarged hilar shadow or small patchy shadows (Figure 7). Negative LUS results may be due to a small amount of fluid in the lung [14]. The result indicates that the sensitivity of LUS may be lower in the early phase of HAPE.

LUS also showed an improved negative diagnostic value for HAPE. The specificity of LUS had no significant difference compared with the specificity of moist rales or CXR.

B-lines are a sign of pulmonary edema but also a sign of interstitial pneumonia and severe pulmonary fibrosis, which may generate diffuse intensive B-lines [4, 15, 16]. Therefore, LUS should be integrated with other clinical information, including symptoms, physical signs, and medical history. In fact, nearly all routine diagnostic methods need to be integrated with other clinical information to arrive at the correct diagnosis. Even typical X-ray signs can lead to a misdiagnosis of HAPE if the findings are not integrated with other medical information [17–19].

We also found high coincidence rate between CXR and LUS results after treatment; the result indicates that LUS is valuable for the surveillance of HAPE. Compared to CXR, LUS is more convenient and faster and is especially suitable for use in emergency departments [20, 21]. LUS is also suitable for use in pediatrics due to the advantage of not incorporating radiation during the imaging process [22].

## 5. Conclusion

To the best of our knowledge, this study is the first to reveal the actual diagnostic value of LUS for HAPE through comparison with auscultation or CXR and this study is the largest study involving the diagnosis and monitoring of HAPE by LUS to date.

The main manifestation of HAPE was multiple B-lines. LUS had a high sensitivity and specificity for the diagnosis of HAPE. Otherwise, LUS showed a favorable surveillance effect for the treatment of HAPE. Thus, we recommend the use of LUS for the diagnosis and monitoring of HAPE.

## 6. Limitations

As it has been mentioned before, B-lines is not the sign presented only in pulmonary edema, but it may also indicate interstitial lung diseases. The good results of LUS in our research are probably related to the high prevalence of HAPE in the population (126/(126 + 22)). Lack of larger sample size is no doubt a limitation of our research. However, according to the past researches, the incidence of interstitial lung diseases combined with acute high-altitude expose history was very rare, only limited to some case reports; meanwhile, accurate diagnosis of these cases can be gotten though comprehensive analysis for history, symptoms, and physical signs [17, 18]. So, even influenced by this limitation, LUS is still a useful tool for the diagnosis of HAPE in most situations.

Due to the restriction of ethics and funds, CT was not performed widely in this study, especially when the LUS and CXR results did not match. We believe that the utilization of CT may enhance the persuasiveness of this research.

The monitoring by LUS during the process of treatment was not performed because of the lack of researchers. The relative research may better reveal the surveillance effect of LUS for HAPE.

## Figures and Tables

**Table 1:** Characteristics of enrolling patients.

	HAPE (*n*=126)	Non-HAPE (*n*=22)	*P* value
General characteristics			
Age (years)	31.09 ± 7.65	31.40 ± 9.73	0.886
Male	119 (90.9)	20 (94.4)	0.000
Heart rate(beats per minute)	108.50 ± 18.82	95.77 ± 10.51	0.000
Systolic arterial pressure (mmHg)	113.79 ± 14.35	122.14 ± 18.34	0.000
Diastolic arterial pressure (mmHg)	75.60 ± 11.61	78.77 ± 14.18	0.000
Time of high altitude exposed (days)	3.14 ± 1.56	3.64 ± 1.89	0.000
Oxygen saturation (%, before treatment)	65.12 ± 7.77	78.05 ± 2.40	0.000
Oxygen saturation (%, after treatment)	88.10 ± 2.17	88.05 ± 1.91	0.479
Clinical characteristics			
Moist rales (positive)	102 (80.95)	3 (13.64)	0.000
CXR (positive)	117 (92.86)	1 (4.55)	0.000
LUS characteristics			
B-lines positive (B-lines score >5)	124 (98.41)	2 (9.09)	0.000
Normal lung ultrasound	2 (1.59)	20 (90.91)	0.000
Other abnormal	0 (0)	0 (0)	
B-line score of HAPE (before treatment)	57.14 ± 42.93	0.64 ± 1.79	0.000
B-line score of HAPE (after treatment)	0.17 ± 1.13	0.00 ± 0.00	0.000

Data are presented as mean ± standard deviation or number (%).

**Table 2:** Characteristics of enrolling patients.

HAPE severity according to HULTGREN grades	
Grade 1 (mild)	3
Grade 2 (moderate)	21
Grade 3 (serious)	93
Grade 4 (severe)	9
Patients of non-HAPE (number %)	22 (14.9)
Bronchitis	1 (0.7)
Acute upper respiratory infection	4 (2.7)
Acute mild high-altitude disease	17 (11.5)
Radiologic score of extravascular lung water of HAPE (before treatment)	
Median (IQR)^*∗*^	39 (29–48)
Range	0–83

^*∗*^Interquartile range (IQR) expressed as the 25th and 75th percentiles.

**Table 3:** Results of LUS, CXR, and auscultation for moist rales before treatment.

	HAPE+	HAPE−
LUS+	124	2
LUS−	2	20
CXR+	117	1
CXR−	9	21
Auscultation+	102	3
Auscultation−	24	19

**Table 4:** Diagnostic value of LUS, CXR, and moist rales to HAPE.

	LUS (95% CI)	CXR (95% CI)	Moist rales (95% CI)
Sensitivity	0.98 (1.01–0.96)	0.93 (0.97–0.88)	0.81 (0.88–0.74)
Specificity	0.91 (1.03–0.79)	0.95 (1.04–0.87)	0.86 (1.01–0.72)
Positive predictive values	0.98 (1.01–0.96)	0.99 (1.01–0.98)	0.97 (1.00–0.94)
Negative predictive values	0.91 (1.03–0.79)	0.70 (0.86–0.54)	0.44 (0.59–0.29)
Positive likelihood ratio	10.83 (64.07–4.56)	20.43 (110.45–2.40)	5.94 (14.29–1.73)
Negative likelihood ratio	0.0175 (0.0993–0.0063)	0.0748 (0.1414–0.0396)	0.2206 (0.2638–0.1194)

**Table 5:** Results of CXR compared with LUS in HAPE patients after treatment.

	CXR+	CXR−	Total
LUS+	2	1	3
LUS−	3	110	113
Total	5	111	116

## Data Availability

The data of the research had been shared in the ResMan research manager (http://www.medresman.org/pub/cn/proj/projectshow.aspx?proj=2051).

## Additional Points

Lung ultrasound (LUS) has proven to be a reliable tool for the diagnosis of pulmonary diseases. However, the actual diagnostic value of LUS for HAPE is still unknown. Our objective was to determine the feasibility of using LUS for the diagnosis of HAPE. Our study got the following results: LUS had higher sensitivity than moist rales and CXR for the diagnosis of HAPE. After treatment, LUS was consistent with CXR in 96.55% of HAPE patients. The results indicate that LUS is a reliable method for the diagnosis and surveillance of HAPE.
